# Quantum Einstein-de Haas effect

**DOI:** 10.1038/ncomms11443

**Published:** 2016-04-29

**Authors:** Marc Ganzhorn, Svetlana Klyatskaya, Mario Ruben, Wolfgang Wernsdorfer

**Affiliations:** 1 Institut Néel, CNRS & Université Joseph Fourier, BP 166, 25 Avenue des Martyrs, 38042 Grenoble Cedex 9, France; 2 Institut of Nanotechnology (INT), Karlsruhe Institute of Technology (KIT), 76344 Eggenstein-Leopoldshafen, Germany; 3 Institut de Physique et Chimie des Matériaux de Strasbourg (IPCMS), CNRS-Université de Strasbourg, 67034 Strasbourg, France

## Abstract

The classical Einstein-de Haas experiment demonstrates that a change of magnetization in a macroscopic magnetic object results in a mechanical rotation of this magnet. This experiment can therefore be considered as a macroscopic manifestation of the conservation of total angular momentum and energy of electronic spins. Since the conservation of angular momentum is a consequence of a system's rotational invariance, it is valid for an ensemble of spins in a macroscopic ferromaget as well as for single spins. Here we propose an experimental realization of an Einstein-de Haas experiment at the single-spin level based on a single-molecule magnet coupled to a nanomechanical resonator. We demonstrate that the spin associated with the single-molecule magnet is then subject to conservation of total angular momentum and energy, which results in a total suppression of the molecule's quantum tunnelling of magnetization.

## Abstract (web summary)

The Einstein-de Haas effect is a manifestation of the conservation of angular momentum, causing a magnetic object to rotate as its magnetization state is changed. Here, the authors demonstrate this effect at the single spin level for a molecular magnet suspended on a nanomechanical resonator.

## Graphical abstract

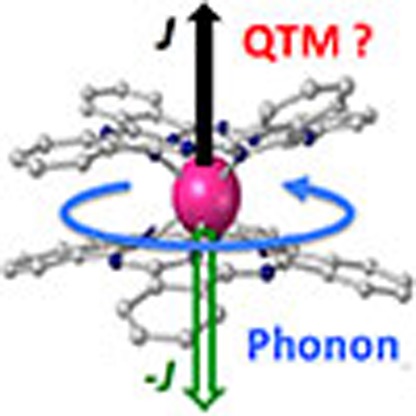

One hundred years ago it has been discovered that a change of magnetization in a macroscopic magnetic object results in a mechanical rotation of this magnet. The effect, known as Einstein-de Haas or Richardson effect, demonstrates that a spin angular momentum in the magnet compensates for the mechanical angular momentum associated with its rotation^1^,^2^,^3^. The experiment is therefore a macroscopic manifestation of the conservation of total angular momentum and energy in eletronic spins. According to Noether's theorem^4^, conservation of angular momentum follows from a system's rotational invariance and would be valid for the ensemble of spins in a macroscopic ferromagnet as well as for an individual spin. It has been recently proposed that single-spin systems would therefore manifest an Einstein-de Haas effect at the quantum level^5^,^6^,^7^.

Here we describe an experimental realization of a quantum Einstein-de Haas experiment, which consists of a single-molecule magnet (SMM) attached to a carbon nanotube (CNT) mechanical resonator. We demonstrate that the spin associated with the single-molecule magnet is then subject to conservation of total angular momentum and energy. At small applied fields, this results in a total suppression of the molecule's quantum tunnelling of magnetization, whereas at higher magnetic field the magnetization reversal occurs via a direct transition between the electronic spin states for a transition energy matching the phonon energy of the CNT mechanical resonator. These findings demonstrate the importance of angular momentum conservation in magnetic nanostructures and are crucial to the field of molecular quantum spintronics.^8^ Indeed, the presented suppression of quantum tunnelling could help to increase the spin lifetime T_1_ of SMMs and other nanomagnets.

## Results

### Single-molecule magnets

A SMM is a nanomagnet, consisting of exchange-coupled magnetic centres, typically a rare earth or transition metal ion, embedded in a shell of organic ligands^8^,^9^. The ligands are designed to promote the molecule's coupling to the environment while protecting and enhancing the ion's magnetic properties. Rare earth-based SMMs can have a large magnetic ground state with a spin *S*>1 and a strong magnetic anisotropy defining the molecule's easy axis of magnetization^10^,^11^. The spin physics can thus be described by a ‘giant spin' Hamiltonian of the form


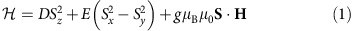


where *S*_*x*_, *S*_*y*_, *S*_*z*_ are the spin components, *D* the axial and *E* the transverse magnetic anisotropy constants (with 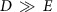), and 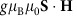 the Zeeman energy associated with an applied external magnetic field. At low temperatures 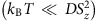, the SMM can be described as a two-level system^10^,^11^ and the magnetization reversal of the SMM can then occur via two different processes^10^,^11^. During a direct transition process (DT), the SMM's magnetization reversal is enabled by the excitation of phonons with a finite energy in the molecule's environment at higher magnetic fields^10^,^12^. Alternatively, the magnetization reversal occurs by quantum tunnelling of magnetization (QTM) between up and down spin polarization at low magnetic fields^10^,^13^. The tunnelling probability of the molecule is given by the magnitude of the tunnel splitting Δ that arises from the transverse magnetic anisotropy component^10^,^13^.

**Figure 1:**
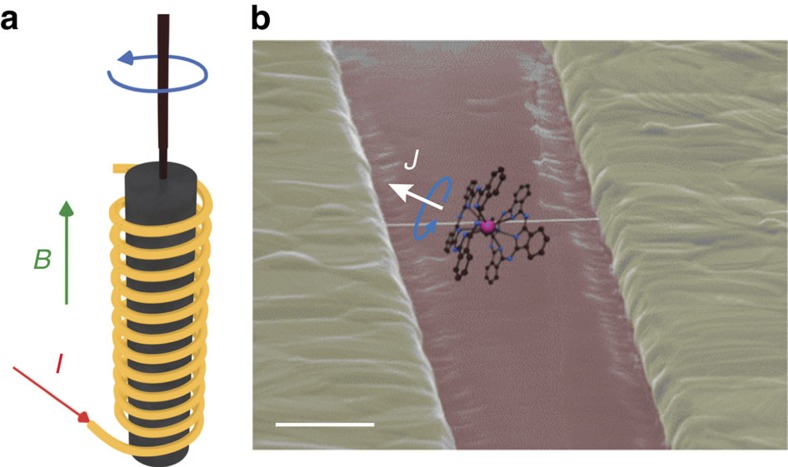
Set-up of the Einstein-de Haas experiments. (**a**) For the classical Einstein-de Haas experiment, a magnetic material is suspended with the aid of a thin string inside a magnetic field coil. When the magnetic field *B* is increased by the application of an electric current *I* through the field coil, the magnetic material is magnetized. In order to keep the total magnetic moment of the magnetic material constant, the latter rotates. This classical Einstein-de Haas effect demonstrates that the spin angular momentum is of the same nature as the angular momentum of rotating bodies as conceived in classical mechanics. (**b**) For the quantum Einstein-de Haas experiment, the false colour scanning electron micrograph shows a suspended carbon nanotube with a local metallic backgate (red) functionalized with a pyrene-substituted bis(phthalocyaninato)terbium(III) (hereafter TbPc_2_) single-molecule magnet (shown as a chemical structure overlaid on the image, not to scale, pyrene omitted for clarity). Due to conservation of the total angular momentum, the magnetization reversal of *J*=6 (white arrow) in a magnetic field results in a rotation of the single-molecule magnet (blue arrow), thus generating a quantized phonon mode in the carbon nanotube nanoelectromechanical resonator. Scale bar, 500 nm.

According to Noether's theorem, both reversal processes have to conserve the total angular momentum as well as energy. For instance, a free SMM that is completely isolated from its environment cannot exchange angular momentum and energy with the latter. Magnetization reversal would be fully supressed in order to conserve total angular momentum and energy^14^. On the other hand, it has been predicted that magnetization reversal can occur in a SMM coupled to a nanomechanical resonator^5^,^6^,^7^. In analogy to a classical Einstein-de Haas effect (Fig. 1a), the magnetization reversal results in a rotation of the SMM to satisfy conservation of angular momentum and energy, and generates a quantized phonon mode in the resonator (Fig. 1b). However, vibrational or rotational modes in nanomechanical resonators carry a non-zero angular momentum and their energy (ℏ*ω*_r_: MHz-GHz (ref. 15)) is typically larger then the tunnel splitting (Δ: kHz (ref. 13)). Consequently, QTM in a SMM coupled to a nanoresonator would be suppressed to conserve both energy and angular momentum^16^,^17^. Hence, magnetization reversal in the SMM only occurs by a direct transition and the excitation of a resonator mode. In this letter, we report on experimental evidence for such a quantum Einstein-de Haas effect and demonstrate that conservation of total angular momentum and energy fully suppresses QTM in a SMM coupled to a carbon nanotube nanoresonator.

### TbPc_2_ single-molecule magnet

**Figure 2:**
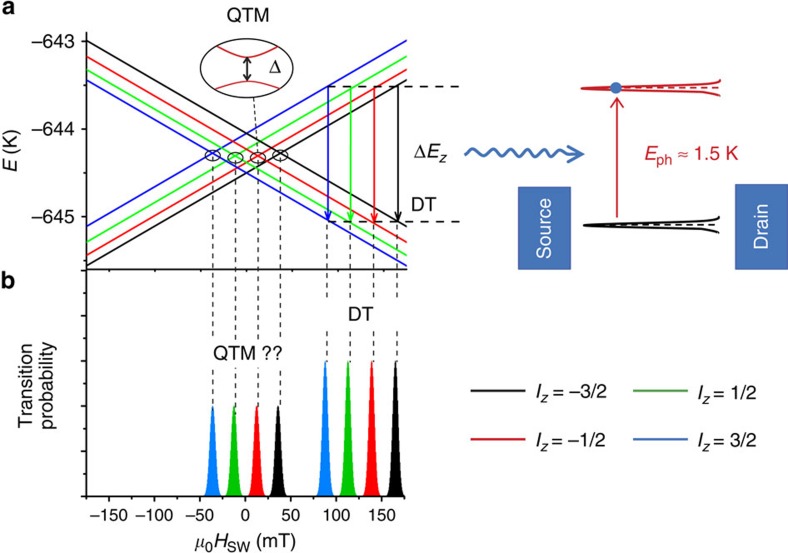
Energy schematics of the quantum Einstein-de Haas experiment. (**a**) Zeeman diagram of the ground-state energy levels of the TbPc_2_ molecule with the magnetic field applied parallel to the easy axis of magnetization. Because of a strong hyperfine interaction with the Tb nuclear spin, the electron's up and down states are each split into four energy levels, labelled with the nuclear spin states *I*_*z*_=±1/2, ±3/2. At higher magnetic field, the magnetization reversal can occur via a direct transition (DT) between the electronic spin states for a transition energy Δ*E*_z_ matching the phonon energy *E*_ph_=1.5 K. Note that, for all transitions, the nuclear spin is preserved and therefore their positions reveal the nuclear spin states. At small fields, off-diagonal terms in the spin Hamiltonian lead to avoided energy level crossings (shown in the inset), enabling in principle tunnelling of the electronic spin. However, QTM is suppressed due to the absence of a phonon mode in the carbon nanotube resonator, which can absorb the angular moment and has in addition a very low energy, being of the order of magnitude of the tunnel splitting Δ∼1 μK. (**b**) Magnetization reversal histograms of the TbPc_2_ molecule coupled to the phonon mode of the carbon nanotube resonator. According to (**a**) one would expect the absence of QTM events (labelled by ‘QTM ??') due to the coupling to the nanoresonator.

The pyrene-subtituted bis(phthalocyaninato)terbium(III) molecule (TbPc_2_) is a rare earth SMM in which the magnetic moment is carried by a single Tb^3+^ ion sandwiched between two organic phthalocyanine (Pc) ligand planes^11^,^12^ (see Methods for synthesis). The TbPc_2_ has a *S*=1/2 radical delocalized over the Pc ligand planes. Due to *π*−*π* interaction, this radical can easily hybridize with the π-electrons of any form of *sp*_2_-carbon without affecting the magnetic properties of the Tb^3+^ ion^11^,^12^,^18^,^19^. The highly anisotropic 4*f* shell of the Tb^3+^ ion and its intrinsically strong spin-orbit coupling result in a magnetic ground state of *J*=6 and a pronounced uniaxial magnetic anisotropy (Fig. 2a). The ground-state doublet *J*_z_=±6 is separated from the excited states by several hundreds of Kelvin, which makes the TbPc_2_ an Ising-like spin system at low temperature (*T*<5 K) and small magnetic field (*B*<10 T)^11^,^12^. A strong hyperfine interaction with the nuclear spin *I*=3/2 of the Tb^3+^ ion splits the ground state doublet *J*_z_=±6 into four states each (Fig. 2a). Finally, the ligand field generates a small transverse magnetic anisotropy resulting in avoided level crossings (black circles in Fig. 2a)^11^,^12^.

### Magnetization reversal of a SMM

According to the Landau-Zener-Stueckelberg-Majorana formalism^20^,^21^,^22^,^23^, the magnetization reversal will occur via QTM at one of the four avoided level crossings around zero field (black circles in Fig. 2a), with a probability *P*_QTM_ given by


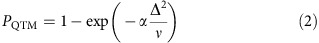


with the tunnel splitting Δ, *ν*=d*H*/d*t* the magnetic field sweep rate and *α* a coefficient specific to the SMM. Alternatively, the spin reversal occurs via a direct transition with a probability 1−*P*_QTM_ at a larger value of magnetic field, the so-called switching field *μ*_0_*H*_SW_. As demonstrated in ref. 12, the switching field *μ*_0_*H*_SW_ is determined by the energy of the phonon(s) involved in the process.

Owing to conservation of total angular momentum, the magnetization reversal from *J*_*z*_=6 (white arrow in Fig. 1b) to *J*_*z*_=−6 of a TbPc_2_ SMM grafted to a suspended carbon nanotube (see Methods for sample fabrication) results in a rotation of the SMM around its magnetic easy axis (blue arrow Fig. 1b), thus generating a quantized longitudinal phonon mode in the carbon nanotube resonator. We demonstrated in ref. 12 that the magnetization reversal in such a supramolecular spintronic device can occur via direct transition from the nuclear spin states of the Tb^3+^, for a transition energy Δ*E*_*z*_ matching the phonon energy *E*_ph_=1.5 K (Fig. 2a,b). As we will demonstrate in the following, the probability for QTM on the other hand is fully suppressed in the absence of a phonon mode, which can absorb the angular moment and has in addition a very low energy, being of the order of magnitude of the tunnel splitting Δ∼1 μK (Fig. 2a,b).

### Quantum Einstein-de Haas experiment

**Figure 3:**
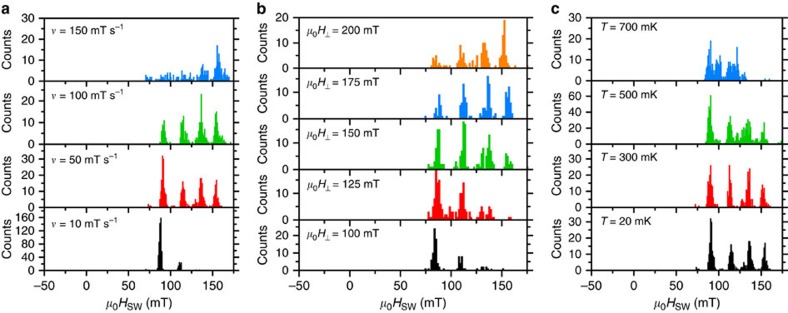
Magnetization reversal histograms of the quantum Einstein-de Haas experiment. Histograms of the swichting fields *μ*_0_*H*_SW_ for several (**a**) field sweep rates, (**b**) transverse magnetic fields and (**c**) temperatures. Each histogram corresponds to 200 field sweeps from negative to positive magnetic fields. No QTM is observed around zero magnetic field, resulting in a 100% probability for a magnetization reversal via a direct transition of the electronic spin state of the Tb^3+^ ion, which depends on the four nuclear spin states. Note that if the SMM had tunnelled at small fields without an observable conductance jump, it could not make a direct transition because it would already be in the ground state.

Using an electronic readout (see Methods), we study the magnetization reversal of the TbPc_2_ SMM coupled to the carbon nanotube resonator as a function of the magnetic field sweep rate *ν*, the transverse magnetic field component *μ*_0_*H*_⊥_ and the temperature *T*. Figure 3a shows histograms of the switching fields *μ*_0_*H*_SW_ extracted from 200 back and forth magnetic field sweeps, for four different magnetic field sweep rates *ν*. According to equation (1), one can alter the QTM probability by tuning the sweep rate of the magnetic field. However, for sweep rates *ν* ranging from 10 to 150 mT s^−1^, we observe a 100% probability for a magnetization reversal via direct transitions, whereas a zero probability for a reversal via QTM is found.

Furthermore, it was shown in Mn_4_ SMM crystals^24^ and individual TbPc_2_ SMM integrated in carbon nanotube transistors^13^ that a transverse magnetic field modifies the tunnel splitting Δ and thus the magnet's QTM probability *P*_QTM_. Specifically, a transverse magnetic field of a few 100 mT can modify the QTM probability *P*_QTM_ of an individual TbPc_2_ by a factor of 2–3 (ref. 13). Figure 3b shows the switching field distribution of the TbPc_2_ SMM coupled to the carbon nanotube resonator for transverse magnetic fields *μ*_0_*H*_⊥_ ranging from 100 to 200 mT. We find a probability for QTM equal to zero with a 100% probability of magnetization reversal of the SMM via direct transitions (Fig. 3b).

Finally, we consider the magnetization dynamics of the TbPc_2_ SMM coupled to the carbon nanotube resonator as a function of temperature. As a matter of fact, additional phonon modes can be excited in the carbon nanotube resonator at higher temperatures and it has been demonstrated that tunnelling processes in a SMM can also be thermally activated^25^. However, the total suppression of QTM persists for temperatures up to *T*=700 mK, whereas direct transitions of the electronic spin of the TbPc_2_ SMM can still occur with a dependance on the nuclear spin state (Fig. 3c).

**Figure 4:**
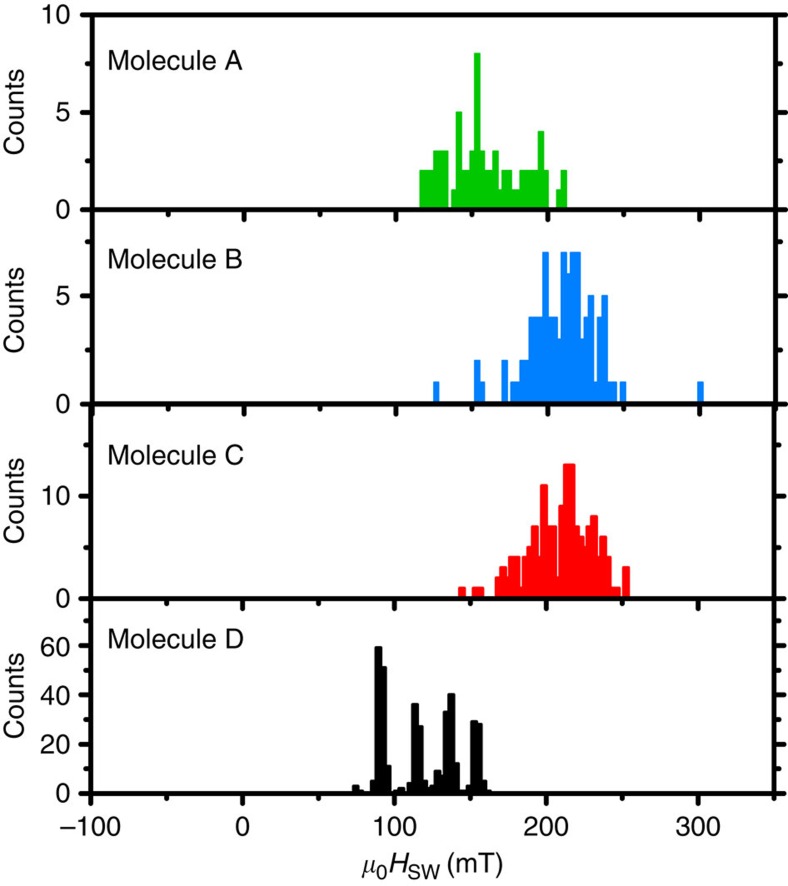
Magnetization reversal histograms of the quantum Einstein-de Haas experiment on different TbPc_2_ molecules. The histograms are recorded at *T*=20 mK and a magnetic field sweep rate of 50 mT s^−1^. It should be noted that the nuclear spin states cannot be resolved in molecules A–C, which can be attributed to the presence of strong fluctuating magnetic fields resulting in linewidth broadening, to weak interaction of the nuclear spin with its environment, or to a small quality factor of the CNT resonator. No QTM is observed around zero magnetic field, but a 100% probability for a magnetization reversal via a direct transition of the electronic spin state of the Tb^3+^ ion.

These findings can thus be considered a direct manifestation of the conservation of angular momentum and energy, that is, the quantum Einstein-de Haas effect, which fully suppresses QTM of the TbPc_2_ in the absence of a phonon mode in the carbon nanotube resonator, capable of absorbing the change of angular momentum Δ*J*_z_=12 and the energy Δ∼1 μK associated with the QTM process in TbPc_2_ SMM. Indeed, we only observe a quantized longitudinal stretching mode phonon in the carbon nanotube resonator, with an energy *E*_long_=1.5 K much larger than the tunnel splitting Δ∼1 μK. In general, the expected energy for a bending-mode phonon (*E*_bend_∼0.01 K, ref. 26), longitudinal stretching-mode phonon (*E*_long_∼1 K) or a radial breathing-mode phonon (*E*_RBM_∼500 K, ref. 26) in carbon nanotube resonators is orders of magnitude larger than the tunnel splitting Δ. Consequently, QTM should be always suppressed in TbPc_2_ SMM coupled to a carbon nanotube resonator. Figure 4 shows the magnetization reversal of four different TbPc_2_ molecules coupled to different carbon nanotube resonators. Indeed we observe the full suppression of QTM on all four molecules. The direct transition of each molecule occurs with a probability of 100%, that is, it cannot have made a tunnel transition at small fields. However, the transitions of the four samples occur at different switching fields *μ*_0_*H*_SW_, which is related to different energies of the involved phonon mode. It should be pointed out that we do not observe stochastic fluctuations between *J*_*z*_=6 and *J*_*z*_=−6, indicating that we are not resonantly exciting the phonon mode.

## Discussion

The robustness of the observed phenomena towards external manipulation further corroborates theoretical predictions of a quantum Einstein-de Haas effect, that is, that single-spin dynamics such as quantum spin tunnelling are ultimately governed by conservation of total angular momentum. Note in particular that all kinds of structural defects of the device or molecule would enhance the tunnel rates because any symmetry breaking induces transverse anisotropy terms in the spin Hamiltonian, which increases the mixing of the spin wave functions. This results in an increase of the tunnel splitting and thus of the tunnel rates.

In conclusion, we can confirm theoretical predictions of a quantum Einstein-de Haas effect, which states that total angular momentum and energy have to be conserved for individual spins^5^,^6^,^7^. The effect manifests itself as a total suppression of QTM in a SMM coupled to a carbon nanotube nanoresonator and is robust against changes in temperature, magnetic field or phonon energy of the nanoresonator. It was recently predicted that such a quantum Einstein-de Haas effect effectively screens the molecular spin against quantum fluctuations, therefore enabling the coherent spin manipulation on a single-phonon level^16^.

## Methods

### Nanofabrication

The suspended carbon nanotube resonators are built in an ultraclean, bottom-up fabrication process. For this purpose, a 1-μm-wide metallic local gate is patterned by optical deep ultraviolet lithography and subsequent e-beam evaporation of Mo (20 nm) on a degenerately p-doped silicon wafer with a 300-nm-thick layer of thermal SiO_2_. A layer of 100 nm of Al_2_O_3_ is then deposited by atomic layer deposition. Using optical deep ultraviolet lithography and e-beam evaporation of Mo (20 nm) and Pt (160 nm), source-drain electrodes are aligned above the local gate. Suspended carbon nanotubes are finally grown by chemical vapor deposition (CVD) at 800 °C from a CH_4_ feedstock and Fe/Mo catalyst spots patterned on the source-drain electrodes next to the junction. The lengths of the carbon nanotubes are estimated to a range of 800–900 nm (ref. 12). In a second step, pyrene-substituted TbPc_2_ SMMs are synthesized by a divergent multistep protocol utilizing two different phthalocyaninato lithium salts and [Tb(acac)3*2H_2_O] (acac=acetylacetonato) in 1:1:1 ratio^18^. Finally, the TbPc_2_ powder is dissolved in a solution of dichloromethane and drop-casted onto the sample. The droplet is subsequently dried in a critical point dryer, to avoid destruction of the suspended carbon nanotubes through capillarity effects^12^.

### Detection of SMM magnetization reversal

Magnetic field sweeps are performed along the TbPc_2_ easy axis from negative to positive magnetic field values and back, while monitoring the differential conductance in the carbon nanotube resonator. The magnetization reversal of the Tb^3+^ ion in a sweep translates as a jump in the nanotube's differential conductance^10^,^12^. The corresponding magnetic field, the so-called switching field *μ*_0_*H*_SW_ of the TbPc_2_ SMM, is then extracted from each sweep. Upon repeating the measurement one can construct magnetization reversal histograms as shown in Figs 3 and 4.

The magnetization reversal measurements are carried out in He_3_/He_4_ dilution refrigerator with a base temperature of 20 mK. The refrigerator is equipped with two orthogonal magnetic fields coils, generating up to 1.4 T and up to 0.5 T in the plane of the sample, with a maximum sweep rate of 250 mT s^−1^. One can orient the field direction with respect to the SMM's easy axis by probing the magnetization reversal at different magnetic field angles^12^.

## Additional information

**How to cite this article:** Ganzhorn, M. *et al.* Quantum Einstein-de Haas effect. *Nat. Commun.* 7:11443 doi: 10.1038/ncomms11443 (2016).
